# Experiences and views of Dutch general practitioners regarding physician-assisted death for patients suffering from severe mental illness: a mixed methods approach

**DOI:** 10.1080/02813432.2021.1913895

**Published:** 2021-07-09

**Authors:** Rosalie Pronk, Nieke P. Sindram, S. van de Vathorst, D. L. Willems

**Affiliations:** a Department of General Practice, Medical Ethics Section, Amsterdam UMC, Academic Medical Centre, Amsterdam, The Netherlands; b Department of Medical Ethics and Philosophy, Erasmus Medical Centre, Rotterdam, The Netherlands

**Keywords:** Physician-assisted death, psychiatry, General Practitioners, psychiatric patients

## Abstract

**Background:**

In the Netherlands, physician-assisted death (PAD) is allowed under certain conditions. Patients who suffer from mental illnesses are not excluded from this practice. In 2018, general practitioners (GPs) performed 20 out of a total of 67 cases of EAS for psychiatric suffering

**Objective:**

More insight into GPs’ experiences and views with regard to PAD in psychiatry.

**Design:**

The data for this study were obtained through a survey amongst 500 randomly selected Dutch GPs and by in-depth interviews with 20 Dutch GPs.

**Setting:**

A survey study and in-depth interviews.

**Subjects:**

Dutch GPs.

**Results:**

86 out of 101 GPs found it conceivable to perform EAS in case of somatic disease, and 51 out of 104 GPs found it conceivable in the case a patient suffered from a mental illness only. The main reason given for refusing an PAD request was that the criteria of due care were not met. Reasons for supporting psychiatric PAD related to responsibility, self-determination, compassion, fairness, and preventing suicide. Reasons for not supporting psychiatric PAD were related to the scope of medicine, a perceived lack of experience, uncertainties regarding the criteria of due care and life-expectancy.

**Conclusion:**

GPs are less likely to perform PAD for suffering from a mental illness, compared to somatic suffering. Some GPs apply an extra criterion of ‘life-expectancy’ in case of PAD for suffering from a mental illness. Refusing PAD based on a long life expectancy keeps open the possibility of recovery, but may also just prolong the suffering and add to the unbearableness of it.

**KEY POINTS**
- Currently, there is no qualitative research on what the views are of general practitioners regarding the subject of physician-assisted death (PAD) for patients suffering from severe mental disorders.
- General practitioners are less likely to consider a request for physician-assisted death by a patient suffering from a psychiatric disorder, compared to somatic suffering. Reasons for supporting psychiatric PAD related to responsibility, self-determination, compassion, fairness, and preventing suicide.
- Reasons for not supporting psychiatric PAD were related to the scope of medicine, a perceived lack of experience, uncertainties regarding the criteria of due care and life-expectancy.
- Significance for the reader: Although allowed in the Netherlands, PAD in case of severe mental suffering remains a controversial topic. We need in-depth information about the actual practice of it to have an informed debate with regard to this subject.

## Introduction

Physician-assisted death (PAD) is a regulated practice in the Netherlands. The Dutch Termination of Life on Request and Assisted Suicide Act (Wtl) is in force since 2002 and allows physicians to perform PAD if (and only if) certain conditions, the criteria of due care, are met. PAD is neither a right of the patient nor a duty of the physician, as it is not considered a ‘normal medical act’. Physicians are free to refuse a request for PAD, and indeed do so for a variety of ethical, psychological and personal reasons [1,2].

In 1994, the Dutch Supreme Court ruled that there are no grounds to exclude patients who suffer from a mental illness from the option of PAD [3]. The number of requests for PAD by patients suffering from a mental illness has risen since, and is estimated to have increased from 320 in 1995 to 1100 in 2016 [4]. The number of performed cases also increased, from zero in 2002 to 67 in 2018 [5]. This shows that although the numbers are rising, the vast majority of requests are denied. A recent study of patients at Expertisecentrum Euthanasie showed that patients who requested PAD on grounds of suffering from a mental illness were predominantly female, and suffered from depression [6]. The majority of these patients had more than one psychiatric diagnosis, and were between the ages of 41–60 [6]. An analysis of granted PAD requests for psychiatric suffering between 2015 and 2017, showed that 77% on grounds of suffering from a mental illness regarded women, and 51% were between the ages of 50 and 70 [7].

The support for psychiatric PAD is higher in the general public than among physicians [8,9]. Physicians in general are less likely to perform PAD for a patient exclusively suffering from a psychiatric disorder [2,4]. The percentage of psychiatrists who can conceive of ever performing PAD on a patient suffering from mental illnesses decreased over the years [4]. A previous study showed that GPs find it less conceivable to perform PAD when a patient suffers from a mental illness, compared to for example suffering from cancer [2].

Various issues arise regarding PAD for psychiatric suffering: the ‘criteria of due care’ can be more difficult to interpret in case of psychiatric illness compared to somatic suffering. It can be harder to establish whether the patient’s death wish is part of the mental illness, or not [10,11]. The capacity of the patient to understand her situation and make decisions regarding her treatment may be impaired [12]. Patients who are depressed may, for example, hold the unjustified belief that chances of recovery are minimal [13]. The second criterion of the Dutch euthanasia act, stating ‘suffering must be unbearable and without prospect of improvement’, may also be difficult to apply. The diagnostic and prognostic uncertainties that come with mental illnesses could make it difficult to establish whether a patient has a reasonable chance to recover [11,13]. Another issue that is mentioned in the literature is the vulnerability of patients suffering from mental illnesses, and their need for protection [11,14,15]. A final concern regarding physician-assisted death in psychiatry relates to the concept of hope. It has been argued that discussing the option of assisted suicide with a patient suffering from a mental illness might provoke feelings of desperation and demoralization (a loss of hope) in the patient, which could lead to unjustified beliefs about the impossibility of recovery [16,17].

Dutch GPs also receive and perform requests for EAS from patients suffering from mental illnesses. In 20 of the 67 cases in which a patient suffering from a mental illness received PAD in 2018, the GP was the notifying physician [18]. This study was set up because there is an increase in EAS requests of patients suffering from mental illnesses [4], and we do not know GP’s experiences and views on the subject matter. What, for instance, are their reasons for supporting or rejecting the possibility of PAD in case of psychiatric suffering? Therefore, we aimed to answer the question: what are Dutch GPs’ experiences with and views on PAD in case a patient suffers from a mental illness?

## Methods

### Design and data collection

We sent out questionnaires to 500 randomly selected Dutch GPs. We obtained the addresses from a national databank of registered physicians (IMS Health), that works in accordance with the national privacy act (AVG act). The inclusion criterion was that the GP had been working in patient care for the past year.

In October 2018, GPs received a four page questionnaire with questions on their experiences with EAS in psychiatry (see Supplementary survey 2). The questionnaire was similar to the one that was sent out to 500 psychiatrists as part of the Third Evaluation of the Dutch Euthanasia Act [4]. GPs were asked for their experiences with and their opinion on PAD: would they find it conceivable to ever perform PAD for a patient suffering from a mental illness, and, did they, in the year prior to receiving the questionnaire, have experience with a request for PAD by a patient suffering from a mental illness? The data were obtained from October 2018 until February 2019. One reminder was sent during that period of time.

We also interviewed 20 GPs (9 women and 11 men) from September 2018 until February 2019, to obtain more in-depth information about the views of the GPs. The interviews were explorative in nature, and guided by a topic list that included more topics than reported in this paper. The questions were formulated in an open way, to provide enough opportunity for the physicians to talk about their experiences and views. From the richness of data we had to choose, and chose what we believed to be the most relevant data. The interviews were conducted by two researchers: R. P. (PhD student) and N. S. (Master student). Interviews lasted approximately 1.5 h, and were held at the GPs location of choice. An informed consent-form that emphasized the voluntary and confidential character of participation was signed before each interview. All participants agreed on the use of an audio-device, which was kept at the Amsterdam UMC at a place only accessible to R. P. All interviews were transcribed verbatim by a third party, who signed a confidentiality agreement. Data saturation was reached, as no new information came up during the last interviews. No repeat interviews were carried out.

### Respondents

We selected the respondents in various ways: on the basis of their replies to the questionnaire, through the network of the Amsterdam UMC, by addressing physicians following a mental healthcare training (kaderopleiding GGZ) and through snowball-sampling. We aimed for a variety in gender, working area, and views on psychiatric PAD. We interviewed 11 men and 9 women, coming from rural areas, smaller and bigger cities in the Netherlands. On the basis of the questionnaire, we selected GPs who were opposed to psychiatric EAS and those who were open to the option or performed PAD in case of psychiatric suffering.

### Data analysis

The questionnaire was analyzed using IBM SPSS Statistics 25 (SPSS Inc., Chicago, IL). We used descriptive data analysis to obtain the results.

Out of the 20 interviews, 19 were analyzed as one interview was lost because of technical difficulties. After coding and discussing the first two interviews together, RP and NS both analyzed all the interviews separately with the use of MaxQDA 2018 and discussed them afterwards. We coded inductively, by developing codes, code trees, and identified overarching themes. After the analysis, the results were discussed by both researchers and with the supervising researchers (D. W. and S. V.).

## Results

### Response rate

Out of the 110 GPs who responded to the survey, 108 met the eligibility criteria. The response rate was 22%. Two GPs had not been working in patient care during the past year. We structured the quantitative data according to the results from the qualitative study.

#### What are the GPs experiences with and views on PAD for patients suffering from mental illnesses?

Table 1 shows the experiences that the GPs had with PAD requests for patients suffering from a mental disorder (see Table 1). The table shows that a significant number of GPs had ever been asked to assist in a patient’s death, and that most requests are denied because the criteria of due care were considered not to be met.

**Table 1.** EAS requests from patients with a psychiatric disorder, questionnaire data.

	Yes *N*=	No *N*=	*N*=
*Has a patient ever asked you to assist with his or her suicide in the foreseeable future?* (*n* = 106)	43	63	
*Have you ever provided assistance in suicide to a psychiatric patient?* (*n* = 43)	3	40	
*Have you ever refused a request for assistance in suicide from a psychiatric patient?* (*n* = 43)	37	6	
*What was the reason for the refusal of this request?* (*n* = 37) * *multiple answers possible*			
I never perform assisted suicide			6
I never perform assisted suicide in case of a psychiatric patient			7
Did not meet the legal criteria of due care			19
Personal objection specifically related to this case			8
Other			3

Table 2 shows their answers on the conceivability of performing PAD in case of somatic suffering only, in case of somatic *and* mental suffering, and in case of only mental suffering (see Table 2). The table shows that most GPs would consider performing PAD in case the patient suffers from a somatic illness and when a patient suffers from a somatic *and* mental illness. Approximately half of the GPs would consider performing PAD when a patient suffers solely from a mental illness.

**Table 2.** Conceivability of performing a request for assisted suicide.

	Yes *N*=	No *N*=
*Do you find it conceivable that you will perform assisted suicide in case of a patient with a somatic illness?* (*n* = 101)	86	15
*If not, would you refer the patient to another physician (who may possibly grant the request)?* (*n* = 15)	15	**–**
*Do you find it conceivable that you will perform assisted suicide in case of a patient with a somatic* and *psychiatric illness?* (*n* = 102)	81	21
*If not, would you refer the patient to another physician (who may possibly grant the request)?* (*n* = 19)	19	**–**
*Do you find it conceivable that you will perform assisted suicide in case of a patient with a psychiatric illness?* (*n* = 104)	51	53
*If not, would you refer the patient to another physician (who may possibly grant the request)?* (*n* = 52)	51	1

##### Reasons for being in favor of the possibility of psychiatric PAD

The interviews showed that the GPs had multiple reasons for supporting the possibility of PAD in psychiatry. These arguments related to responsibility, self-determination, compassion, fairness, and preventing suicide.

Some GPs indicated that they felt responsible for their patients, and wanted to look after their interests. For some this was a personally felt responsibility, whereas for others this meant a responsibility connected to their profession:

[R: Yes, I actually think that if I stand beside my patient as a doctor, then I have to protect their interests. Of course, how far do you go with that? But in my case, it goes as far as helping someone to die in case life is unbearable.

I: Whether that is on somatic grounds or psychiatric grounds?

R: Yes, if I can empathize with it] (R16)

In the survey, the GPs were asked whether they believed everyone has the right to self-determination in respect to his or her own life. 56 out of 105 agreed with this statement, 19 disagreed and 30 GPs gave a neutral answer.

In the interviews, respondents indicated that patient self-determination was an important reason for them to be in favor of psychiatric PAD:

[I: Could you tell me something about how you view euthanasia or physician-assisted suicide in case of a psychiatric patient?

R:Well, in believe that I am very liberal in general, not in the political sense of the word, but that I find it very important that a human being, if he is capable, should be able to make his or her own choices. In that sense, euthanasia in psychiatry is just as normal as euthanasia for other reasons, and a euthanasia request of a patient is justified if a patient asks for that well-considered, for whatever reason.] (R4)

Respondents mentioned in the interviews that they could empathize with the suffering and that it was meaningful for them to be able to offer a relieve to that suffering:

[I: Could you tell me a little bit more about when you would find it conceivable that you would cooperate with a psychiatric patients’ euthanasia request?

R:You can imagine that if you see somebody that often, see how difficult his life is, how much they are suffering, that you at some point just grant somebody that it can stop. That you saw them for so long, that you just grant it to them…that it is better for them yes.] (R17)

Many GPs compared patients with psychiatric diseases to patients with somatic diseases. Some highlighted the differences, but there were also GPs who emphasized the similarities between the two groups:

[I think my general attitude is fairly liberal, so I can understand that, yes. I see it as sort of a chronic illness, and just as with many chronic illnesses, you can have a wish for euthanasia as a patient. And psychiatry in itself does not mean that you cannot oversee the consequences. I believe that enough psychiatric patients can oversee them, and hence can have a realistic wish for that.] (R12)

In the questionnaire survey, the GPs were asked to give their opinion regarding the statement that PAD is an acceptable option to prevent suicide. The GPs proved to be divided over this matter: 38 out of 103 agreed with the statement, 31 disagreed, and 34 gave a neutral answer.

In the interviews, some respondents wished they could have prevented the suicide of patients by providing PAD:

[I: What is your opinion about physician-assisted suicide in psychiatry? Do you believe that it [i.e. psychiatric disorders] can provide a justified reason…?

R: Yes, I do think so. I have experienced some cases, from very nearby, of patients who suffered from a psychiatric condition who ended their lives. Also in the river across from here, a hundred meters from my house. And then I thought to myself, what a misery, how could it have ever come this far. Why couldn’t I have helped this woman?(…) So I think in some cases, those lives [i.e. of psychiatric patients] are without prospect of improvement and the patient suffers unbearably] (R16)

##### Reasons *for* not *being in favor of the possibility of psychiatric PAD*

In the interviews, some GPs indicated that they were of the opinion that ending a patient’s life does not fall within the scope of medical practice, as the medical profession is concerned with keeping patients healthy and alive:

[On the other hand, killing someone is not a medical act. So the whole euthanasia-issue, should that be in the hands of doctors? Why? We are more involved in life, and keeping life as optimal as possible.] (R14)

Respondents also mentioned that they viewed mental suffering as existential suffering. They indicated that relieving existential suffering does not fall within the scope of medical practice and that doctors do not have the expertise to evaluate existential suffering:

[And I believe we as doctors should stay away from existential suffering, because existential suffering is of all times. We have to relate to that. There is nothing medical about it, so we should not make it medical.] (R 11)

Some respondents believe that ending the life of a patient suffering from a mental illness could fall within the medical realm, but indicated that it is the task of a psychiatrist, and not of a GP.

[I don’t think this should be the task of the GP, because it is about complex psychiatric problems. It is not about a seasonal depression or a relationship crisis. This is about a serious deep wish that people have. That belongs to psychiatry.] (R6)

Respondents mentioned that they felt not at ease with handling a request from a patient suffering from a mental illness, because they lacked the experience:

[It is so far from the diagnoses that normally lead to a euthanasia request, that it is a shady area for me. I don’t feel at home with it. I think that has to do with the lack of experience I have in treating severe psychiatric patients. I refer them all]. (R16)

[You know, a GP knows a little about a lot. And the psychiatrist knows a lot about a little, just as any specialist. And I know for sure that I have big gaps in knowledge when it comes to assessing psychiatric patients.] (R3)

Respondents indicated that psychiatrists have more knowledge on the interpretation of the criteria of due care in case of suffering from a mental illness, on mental competence, on how to differentiate between a pathological and non-pathological wish to die, and on possibilities for improvement:

[I would not be able to assess whether there is no prospect of improvement. For that, in case I would consider performing euthanasia, I would consult a psychiatrist to assess the mental competence and on whether there is no prospect of improvement.] (R6)

However, certain advantages of GPs compared to psychiatrists were also mentioned.

GPs argued that they have more room to be empathetic to the situation of the patient, and because they often know the patient for a very long time, including their social context and background.

In general, the GPs favored working together with psychiatrists during the assessment of the request:

[I do feel competent, but I believe you should do it together, because a psychiatrist has to tell you what has happened and how it helped. You have to know whether you’ve missed something. So, I don’t think you should do it by yourself, it is a collaboration.] (R17)

Table 3 shows how GPs reacted to various statements about the evaluation of the criteria of due care.

**Table 3.** Statements.

	Agree *N*=	Neutral *N*=	Disagree *N*=
it is impossible to assess whether a psychiatric patient’s suffering is unbearable and without prospect of improvement’ (*n* = 104)	15	35	54
it is never possible to establish whether a wish to die is ever well-considered (*n* = 104)	12	34	58
it is possible to establish whether the wish to die is the part of the underlying pathology (*n* = 105)	21	36	48

In the interviews, some respondents expressed concerns related to uncertainty about the criteria of due care, as they told us that one can never know for certain whether a patient can get better:

[And sometimes those people recover. And if you open up the possibility for euthanasia, they never have that option again. We cannot judge that, we are not God.] (R11)

Also, interviewees found it difficult to determine whether a patient’s suffering from a mental illness is unbearable or not, in comparison with somatic suffering. Reasons provided were that the suffering is not visible (as it often is in case of somatic suffering), it is less objectively measurable than somatic suffering, and the suffering is harder to empathize with because of a lack of psychiatric susceptibility of the doctor:

[I: What about the unbearableness, do you consider it enough if the patient says ‘this is unbearable for me’ and you see that it is, is that enough? Or do you really need to be able to empathize with it?

R: Well, when he says it and you see it, I think that is already a lot, that heads towards empathizing. I myself do not have any psychiatric susceptibility, or sombreness, so I will never be able to empathize with that, I will never feel that.] *(R12)*

Some interviewees indicated that the life-expectancy of a patient is a relevant factor in being opposed to the idea of psychiatric PAD. Respondents mentioned that the relatively long life expectancy of patients suffering from mental illnesses, compared to somatic patients, made them more hopeful regarding possibilities for recovery.

[The longer the life expectancy, the greater the chances are, that is my hope, that things will work out. Either intrinsic or extrinsic, that something can be done so that it can work out with that person.] (R13)

Also, the fact that a person’s life is significantly cut short, and that a person has a whole life ahead of them was considered to be relevant:

[Well, because I have the idea that in general with psychiatric complaints, psychiatric problems, it is very hard to determine whether someone will get better or not. And besides that, her age played an important part, because she has –in my eyes- a whole life ahead of her. That is a different situation than someone with metastasised cancer who doesn’t get better anymore.] (R9)

This GP mentioned that ending the life of a young person feels unnatural to him:

[And I can imagine that in case of a very young patient, I would find that very difficult. It feels unnatural to kill a young person. So, I would be troubled by that, yes, that plays a role in case of psychiatric patients.] (R15)

## Discussion

### Summary

This study was set up to gain insight into the experiences and views of Dutch GPs regarding PAD in psychiatry. We combined two studies to obtain these results: a survey-study and an interview-study. The results from our survey-study show that Dutch GPs find it less conceivable to perform PAD in case of only suffering from a mental illness, compared to cases of PAD with regard to only somatic or combined somatic *and* mental suffering. The most important reason given for not granting a request from a patient suffering from a mental illness is that the doctors felt that they did not meet the legal criteria of due care.

### Strengths and limitations

A strength of this study is that it provides insight into a very controversial practice. To our knowledge, no previous qualitative studies have been performed amongst GPs specifically regarding PAD in case of patients suffering from mental illnesses. Another strength is that we have combined two types of studies to obtain our results, a quantitative and a qualitative study. In that way, we were able to show a broad view of the experiences and considerations of the GPs.

A limitation of the questionnaire is the low response rate (22%), which may have led to selection bias. It might be possible that those with a negative attitude towards PAD were more inclined to respond to our questionnaire, to show their dissatisfaction. Our results are, however, fairly consistent with other studies on the subject, so we have little reason to believe that selection bias may have taken place [2,9].

### Comparison with existing literature

Studies regarding the acceptability of PAD among psychiatrists in the Netherlands show that they also find it significantly less conceivable to perform PAD in case of mental suffering only, and that this has even decreased over the past few years [2,9]. In contrast to this, the overall acceptance of psychiatric PAD among the general public increased, and remained the same or also increased among other physicians [12].

Although the Dutch euthanasia law does not differentiate between somatic and mental illnesses, the GPs do seem to make a distinction. It seems that the physicians are reluctant to perform PAD, while the number of psychiatric patients who request PAD has only gone up [4]. Whether PAD is seen as morally acceptable or not in these cases depends on how one balances different values. On the one hand, there is the value of equality, as an aspect of justice, and this was mentioned explicitly by our respondents [19]. They state that patients suffering from mental illnesses may suffer just like patients with somatic diseases, and hence should also be eligible for PAD. On the other hand, it may be more difficult for physicians to fulfil the legal criteria of due care in case of a request from a patient suffering from a mental illness, as is evidenced by the fact that most requests were rejected for that reason. We do not know whether the physician actually did not meet the criteria in those cases, or whether they had difficulties determining whether the criteria were me. In the interview study, the respondents indicated that they did experience difficulties evaluating the criteria. GPs felt insecure with regard to their own expertise on psychiatry.

Just like the GPs, psychiatrists indicated that they were uncertain about certain aspects of the process, however, their uncertainty regarded the evaluation of the criteria, not their own expertise [4]. Some GPs expressed lack of knowledge about the treatment of mental illnesses and found this to be problematic when it comes to PAD. This is interesting, as we would expect them to also experience a lack of knowledge in case of many somatic diseases, and hence to be equally hesitant to perform PAD in case of somatic suffering. However, this was not indicated by the GPs. It may be the case that the relatively long life expectancy of the patients and the fact that a mental illness is rarely terminal plays a role here. The majority of common PAD cases involve patients with cancer [18], and, in these cases, the patient is very likely to die from the disease within a short period of time. The certainty that these patients will pass away in the foreseeable future seems to diminish the physicians’ fear of misinterpreting the criteria of due care. Some GPs seem to apply an extra criterion of due care, namely that of life-expectancy. On the one hand, they feel that helping a younger patient to die is unnatural, and, on the other hand, they related the life-expectancy to the criterion of ‘no prospect of improvement’.

Although these reasons are understandable and physicians always have the right to refuse a request, we think that one aspect remains underexposed. Patients with mental illness whose suffering is without prospect of improvement do not have a shortened life-expectancy as a result of their condition, which means that in the worst case, their suffering could go on for decades. Although the physicians retrieve hope for recovery from the fact that the patient has a relatively long life-expectancy, this is different for the patient. We know that the thought that the suffering will continue for a long time adds to the unbearableness of the suffering. This is illustrated by the fact that some patients suffering from mental illnesses postpone their actual PAD after they received approval for PAD. It is thought this happens because knowing that there is a way out relieves some of the suffering [20,21]. Refusing PAD on the basis of a long life expectancy keeps open the possibility of recovery, but may also just prolong the suffering and add to the unbearableness of it.

### Implications for research and/or practice

As this study shows that GPs are concerned about their perceived lack of knowledge regarding mental illnesses and their treatment, one implication could be that we would need to create more opportunities for the GPs to receive guidance when evaluating requests for PAD from patients suffering from mental illnesses.

## Supplementary Material

Supplemental Material
