# Supplementary material for: Experiences and views of Dutch general practitioners regarding physician-assisted death for patients suffering from severe mental illness: a mixed methods approach
Source: Scand J Prim Health Care. 2021 Jul 9;39(2):166–73. doi: 10.1080/02813432.2021.1913895 (PMC8293937; doi:10.1080/02813432.2021.1913895)
Supplement: Supplemental Material [file IPRI_A_1913895_SM0226.docx]

**
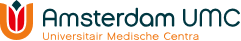
**

**Survey study among general practitioners concerning physician-assisted death in psychiatry**

**Instructions**

**Your data will be treated confidentially**

For most questions you only need to tick one box. If you can tick more than one option for a question, this will be explicitly mentioned.
If you are asked about experiences in the past year, this refers to 12 months prior to the completion of this questionnaire. The questionnaire concerns physician-assisted death. By this we mean both euthanasia and physician-assisted suicide. Please take into account the following definitions when completing the questionnaire:

**Euthanasia:** Deliberate termination of life at the express request of the patient, with the doctor administering the lethal drugs**.**

**Physician-assisted suicide** Deliberate termination of life at the express request of the patient, with the patient administering the lethal drugs himself or herself.

| A. Firstly | | | |  |
| --- | --- | --- | --- | --- |
| A1 | Have you worked as a general practitioner in patient care in the past year? | - Yes | |  |
|  |  | - No 🡪 You do not need to complete this questionnaire. However, please send it back to us in the reply envelope. | |  |
| A2 | 1. How long have you been working as a general practitioner? | years | |  |
|  | 1. How many hours a week do you work as a general practitioner? | hours a week | |  |
|  | 1. Where do you work as a general practitioner?   (one or more answers possible) | - Private practice - Primary setting - General practitioner employed by general practitioner (HIDHA) | |  |
|  |  | - Other, **nl**: | |  |
| A3 | Are you working as a palliative care consultant or at the End-of-Life Clinic (SLK) or as a SCEN-doctor? *(one or more answers possible)* | - No | |  |
|  |  | - Yes, as a SCEN doctor | |  |
|  |  | - Yes, as a palliative care consultant - Yes, at the SLK | |  |
|  |  | - Yes, as a member of a palliative team | |  |
| B. Your experiences with end-of-life decisions | | | | |
| B1 | 1. Has a psychiatric patient ever explicitly asked you for help with suicide in the foreseeable future? (NB where the basis for the request lies exclusively in the psychiatric problems) | - Yes | | |
|  |  | - No 🡪 go on to question **B 3** | | |
|  | 1. Have you ever assisted a psychiatric patient in suicide (NB where the basis for the request lies exclusively in the psychiatric problems). 2. Have you ever refused a request for assisted suicide of a psychiatric patient? (NB where the basis for the request lies exclusively in the psychiatric problems) 3. What was the reason for refusing this request? | - Yes | | |
|  |  | - No - Yes - No 🡪 go on to question **B 2** - I never perform assisted suicide - I never perform assisted suicide in case of a psychiatric patient - Objections of the family and/or relatives - Did not meet the criteria of due care - Personal objections specifically related to this case, nl:      - Other, nl: | | |
| B2 | 1. In the past year, how many psychiatric patients asked you explicitly for assistance with suicide in the foreseeable future? (NB where the basis for the request lies exclusively in the psychiatric problems) | patients | | |
|  |  | - None 🡪 go on to question **B 3** | | |
|  | 1. In the past year, how many psychiatric patients did you assist with suicide? (NB where the basis for the request lies exclusively in the psychiatric problems) | patients | | |
|  | 1. In the past year, how many patients asked you explicitly for assistance with suicide in the foreseeable future, where the suffering was caused by a psychiatric and somatic disorder? | 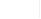 patients  None 🡪 go on to question **B 3** |  | |
|  | 1. In the past year, how many patients did you assist with suicide, where the suffering was caused by a psychiatric and somatic disorder? | patients |  | |
| B3 | 1. Do you find it conceivable that you will perform assisted suicide in case of a patient with a somatic illness | - Yes | | |
|  |  | - No | | |
|  | 1. If not, would you refer the patient to another physician (who may possibly grant the request)? | - Yes | | |
|  |  | - No | | |
|  | 1. Do you find it conceivable that you will perform assisted suicide in case of a patient with a somatic and psychiatric illness? | - Yes | | |
|  |  | - No | | |
|  | 1. If not, would you refer the patient to another physician (who may possibly grant the request)? | - Yes | | |
|  |  | - No | | |
|  | 1. Do you find it conceivable that you will perform assisted suicide in case of a patient with a psychiatric illness? | - Yes - No | | |
|  | 1. If not, would you refer the patient to another physician (who may possibly grant the request)? | - Yes - No | | |

| C. Statements | | | | | | |
| --- | --- | --- | --- | --- | --- | --- |
| Indicate to what extent you agree or disagree with the following statements. This is about your opinion, not what is legally permissible. | | Completely agree | Agree | Neutral | Disagree | Completely disagree |
|  | I have enough knowledge about the euthanasia law |  |  |  |  |  |
|  | Everyone has the right to decide about their own life and death |  |  |  |  |  |
|  | it is impossible to assess whether a psychiatric patient’s suffering is unbearable and without prospect of improvement |  |  |  |  |  |
|  | A psychiatric patient **can** suffer unbearably from their psychiatric disorder |  |  |  |  |  |
|  | Assistance with dying is incompatible with a psychiatric treatment relationship |  |  |  |  |  |
|  | If a patient can end his or her life without help in a non-mutilating way, he may not ask another person for help |  |  |  |  |  |
|  | For psychiatric patients, it is never possible to establish whether a wish to die is well-considered |  |  |  |  |  |
|  | For psychiatric patients, it is never possible to establish whether the death wish is the result of the psychopathology |  |  |  |  |  |
|  | When deciding whether or not to grant the request, the general practitioner does not have to take into account the theoretical possibility that an effective therapeutic intervention may become available in the future. |  |  |  |  |  |
|  | Physician-assisted death by a physician is acceptable in order to prevent suicide |  |  |  |  |  |
|  | In chronic psychiatric patients, the criterion of competence may be less demanding than in other patients |  |  |  |  |  |
|  | Death by consciously stopping eating and drinking can be a good alternative to assisted suicide. |  |  |  |  |  |
|  | I experience pressure from society to comply with a request for assisted suicide of a psychiatric patient. |  |  |  |  |  |
|  | Since the founding of the End-of-Life Clinic, I have been more inclined to refuse requests for assisted suicide of psychiatric patients. |  |  |  |  |  |

| D. Final questions | | | | | |
| --- | --- | --- | --- | --- | --- |
| D1 | 1. What is your gender and age? | Gender: | | - Male | - Female - Other |
|  |  | Age | | years old | |
|  | 1. Are you religious? | - No | | | |
|  | **If yes,** what is your religion? | - Yes, nl: | | | |
| D2 | Have you had training on end-of-life decisions? | - No | | | |
|  |  |  | | | |
|  |  | - Yes, nl: | | | |
| D3 | 1. Are you familiar with the Code of Practice of the Regional Review Committees? | - Yes | - No 🡪 go on to question  **D4** | | |
|  | 1. Have you ever consulted the Code of Practice? | - Yes | - No 🡪 go on to question **D4** | | |
|  | 1. Were you satisfied with the information 2. **If not,** what were you dissatisfied with? | - Yes | - No | | |
| D4 | 1. Are you familiar with the ’guideline for assisted suicide for patients with a psychiatric disorder’ of the Dutch Psychiatric Association? | - Yes | - No 🡪 go on to **End** | | |
|  | 1. **If yes,** have you ever consulted this guideline? | - Yes | - No 🡪 go on to **End** | | |
|  | 1. **If yes,** were you satisfied with the information? 2. **If not,** what were you dissatisfied with? | - Yes | - No | | |

**End**

We would like to invite you for an in-depth interview, in order to get an even better insight into your experiences and views on assisted suicide in psychiatry. If you are open to this, you can indicate this under 'remarks' by writing down your contact details.

**Remarks:**
